# A survey to evaluate knowledge, attitudes, and practices associated with the risk of foodborne infection in a sample of Sicilian general population

**DOI:** 10.3934/publichealth.2022031

**Published:** 2022-05-11

**Authors:** Emanuele Amodio, Giuseppe Calamusa, Salvatore Tiralongo, Federica Lombardo, Dario Genovese

**Affiliations:** Department of Health Promotion, Mother and Child Care, Internal Medicine and Medical Specialties, University of Palermo (Via Del Vespro 133, 90127, Palermo, Italy)

**Keywords:** foodborne disease, KAP questionnaire, household food safety, Sicily, epidemiology

## Abstract

Although foodborne infections contracted at home are frequent diseases worldwide, there is a general lack of information. Main purpose of this cross-sectional study was to evaluate knowledge, attitudes, and practices (KAP) of a sample of the general Sicilian population about the risk of contracting foodborne diseases. It was carried out through a web-based questionnaire to a Sicilian population sample. The questionnaire collected socio-demographic data, health issues, KAP and self-reported diseases. Scores were calculated for summarizing the results. A total of 373 subjects participated into the study. Overall, 65.15% of the participants were females, 48.26% of all respondents were aged between 18 and 29 years and over one-third were students (34.58%). At least one episode of vomiting/diarrhoea in the previous 3 months was reported by 119 respondents. Practices were associated with knowledge (R^2^ = 0.02; p < 0.01) and attitudes (R^2^ = 0.13; p < 0.001) although with low degree of correlation. A lower practice score was statistically significantly associated with both onset of foodborne transmitted infections in participants and among the cohabitants of participants. Our results confirm that foodborne disease can be strongly associated with food handling at home and with unsafe practices. Specific education on food safety could help to reduce the risk but the adoption of good practices of food manipulation is the real key to assure a reduction in food outbreaks in residences.

## 1. Introduction

Foodborne diseases (FBDs) are a group of diseases of infectious or toxic nature caused - or believed to be caused - by the consumption of food or water [1],[2]. FBDs frequently require health care and drug therapies aimed at controlling symptoms that involve mostly the gastrointestinal system, such as abdominal pain, nausea, vomiting and diarrhoea; sometimes, they can give rise to systemic symptoms and complications that rarely can lead to death [3].

According to the World Health Organization (WHO), approximately 600 million people became ill in 2010 after consuming contaminated food. Among these, 420,000 died in the same year, including 125,000 children under the age of 5 [4]. In the United States of America, it has been estimated that about 47.8 million episodes of foodborne diseases, 127,839 hospitalizations and 3037 deaths occur each year; of these, only 20% is attributable to known pathogens [5],[6].

It is, moreover, estimated that 130 million Europeans [7],[8] are affected by episodes of foodborne illnesses every year. This occurrence would mean that about 17% of all European residents can be affected by gastrointestinal disease yearly, and this is confirmed by a recent article estimating a disease burden of 12 Disability Adjusted Life Years (DALYs) per 100,000 subjects [9],[10], roughly corresponding to 54,000 DALYs for 447 million Europeans (estimates of 2020 European Union population) [11].

The known pathogens are responsible for approximately 38.6 million diseases per year. In the USA, known pathogens cause more than 9 million episodes of FBDs, ~55 thousands of hospitalizations and more than a thousand deaths [6]. From the diseases caused by known pathogens, 80% are caused by viruses, 13% by bacteria and 7% by parasites [6].

Numerous studies have examined the potential impact of cross-contamination in relation to home food preparation [12]–[16]. Moreover, it is believed that most (95%) of foodborne illness cases are sporadic [17],[18]. These cases, as well as small outbreaks that originate in the home, usually affect individuals or, in any case, a small number of people with mild symptoms: it is this peculiarity that probably contributes to the underreporting of these FBDs to the competent organs, such as the local and national health authorities. Most people think that foodborne illnesses are mainly associated with foods eaten outside the home while the private home has been found to be the key place where foodborne illnesses are generated [12],[19],[20].

WHO [21] estimated that about 40% of foodborne illnesses were associated with home-cooked food; this percentage is confirmed also by a Brazilian study, which underlines that 38.3% of their study participants had an FBD attributable to the consumption of food within home [22]. Risk factors that are most frequently identified at home are: inadequate forms of cooking, inadequate refrigeration and heat chain processes, wide time interval between preparation and consumption of food, ingestion of raw food [23]–[25].

Studies in many countries have been conducted to evaluate consumer food safety practices, adopting different approaches such as questionnaires and surveys, interviews, and observational studies [14], [26]–[29].

Consumer surveys are used to explore underestimated and/or unrecognized perceptions of risk related to improper behaviour with the prospect of greater food safety at home [30],[31]. Consumer knowledge, attitudes, and practices (KAP) are important to contribute to improved public health programs related to food safety [32],[33].

The main purpose of this study was to evaluate knowledges, attitudes, and practices of a sample of the general Sicilian population regarding their food handling habits. Secondly, we tried to assess potential predictors in the risk of contracting foodborne diseases related to food hygiene at home.

## 2. Materials and methods

### 2.1. Study design

For the analysis of this study purposes, a cross-sectional study was conducted through the administration of a questionnaire to a general Sicilian population sample. The questionnaire was drawn up based on national and international validated questionnaires and on risk factors that are epidemiologically more widespread in the population [35]–[37]. Moreover, we carried out a pilot study upon 36 subjects at a given point and after 20 days, in order to collect their answers in the regards of the survey and assess both reproducibility of the given answers and reliability. The final version of the questionnaire was drafted according to the previous findings and included the following questions:

- Socio-demographic data (4 questions): sex, age, profession, educational qualification. Regarding the Professions field, it has to be noted that several categories have been incorporated under single groups, resulting in the followings: “Construction worker”, “Metal worker”, “Employee”, “Freelancer (lawyer, engineer, consultant, etc)”, and “Other” were included into “Non-health Professional” group; “Housewife”, “Retired”, “Unemployed” were incorporated under “Non-worker” group. The other two categories (“Student” and “Health Professional”) remained unchanged;
- Health issues (2 questions): Chronic Diseases (diabetes, hypertension, chronic bronchitis, chronic heart disease (CHD)) and Gastrointestinal tract Diseases (gastroesophageal reflux disease (GERD), colitis, irritable bowel syndrome (IBS), gastritis);
- Knowledge (6 questions): foods involved in foodborne diseases, factors that determine food diseases, incorrect management of the hot/cold chain;
- Attitudes (8 questions): how to defrost food, how to store both perishable and cooked food;
- Practices (5 questions): cleaning surfaces and utensils after working raw food, washing hands after touching certain foods at risk of cross-contamination and adequate storage of both cooked and raw foods;
- Presence of diseases (2 questions): it has been investigated by asking for the presence of symptoms/signs potentially attributable to foodborne transmitted diseases in the previous 3 months in the participants and in the previous months among their cohabitants, defined as all people living in the same residence as the participants.

*Data collection*

The questionnaire was uploaded to the Google Documents online platform and disseminated via social networks (Facebook, WhatsApp, e-mail) with a “snowball sampling” method by which currently enrolled research participants help recruit future subjects for a study. Data were collected through Google Sheets, an online application in which all the responses of the survey participants were automatically loaded. The full completion of the questionnaire was a necessary condition to send it. The answers of the various users who participated in the questionnaire were collected from 11–11–2020 until 19–01–2021.

At the end of the questionnaire, each user was sent a decalogue of good practices in the field of Food Hygiene, in a logic of Health Literacy [38]; decalogue was composed of ten recommendations to follow to minimize the risk of contracting a foodborne disease. A total of 373 responses were collected at the end of the study.

### 2.2. Statistical analyses

The score was calculated by attributing 1 point to each right question and 0 point to any other answer. The sum of all correct questions allowed us to obtain the knowledges score (between 0 and 6 points), attitudes score (from 0 to 8 points) and practices score (from 0 to 5 points). The sum of the three different scores was used for calculating the overall score ranging from 0 to 19.

Qualitative data have been summarized by frequency and relative frequency (%) whereas quantitative variables have been shown as mean (SD) if normally distributed and median (IQR) for non-normally distributed variables. Shapiro-Wilk test has been performed to verify whether distribution of quantitative variables was normal or not. Kruskal-Wallis test was used to assess the sameness of medians of the four different Scores (Knowledges, Attitudes, Practices; Overall Score) among socio-demographic variables and presence of comorbidities.

To address factors potentially involved into the presence of disease in the study participants, a multinomial logistic regression was used. In this regression, the dependent variables were absence of disease (referent), or the presence of disease one or more than time. Correlation between KAP scores was analysed with coefficient of determination. Moreover, we have implemented a logistic regression analysis to assess variables involved in increasing the risk of foodborne transmitted disease in the co-habitants of the study participants. A backward stepwise approach was used for selecting the final model and a p-value < 0.05 was used as the criterion for inclusion.

Odds ratio (OR) and 95% confidence interval (CI), adjusted for potential confounding, have been calculated as risk indicators for both the multinomial and logistic regression analyses.

All statistical analyses were conducted using the R for Statistical Computing program (R version 4.0.3) and a p-value < 0.05 was considered as statistically significant.

## 3. Results

The characteristics of the study participants are summarized in Table 1. Overall, the majority of the participants were females, nearly half of all the respondents were aged between 18 and 29 years and over one-third were students. In the regards of the educational levels, the most represented groups were subjects with a degree or a secondary school diploma. About 15% of recruited subjects stated that they had a Diagnosis of Chronic Pathology whereas more than one-third stated that they suffer from Gastrointestinal diseases. At least one episode of vomiting or diarrhoea in the previous three months was reported by 86 (one episode) and 33 (more than one episode) of respondents.

Variables involved as predictors of the overall score were reported in Table 2. A statistically significantly higher overall score was observed in respondents aged 70−79 years (p = 0.048), Health Professionals (p < 0.001), and subjects with a university degree (p < 0.001). A statistically significantly lower overall score was found in respondents who did suffer from vomiting/diarrhoea in the previous three months (p < 0.001) and in those living with cohabitants having gastrointestinal symptoms in the previous month (p < 0.001). Table 2 also summarizes the relationship between the investigated variables and each sub-score (knowledge, attitude, and practice score).

As data not shown in the table, we have found that the three sub-scores were correlated the one with each other with the following correlation coefficients: Knowledge – Attitudes (R^2^ = 0.12; p < 0.001), Knowledge – Practices (R^2^ = 0.02; p < 0.01) and Attitudes-Practices (R^2^ = 0.13; p < 0.001).

**Table 1.** Sociodemographic characteristics of the study participants.

N = 373	Number (N)	Percentage (%)
**Age**		
18–29	180	48.3
30–39	59	15.8
40–49	70	18.8
50–59	41	11
60–69	18	4.8
70–79	5	1.3
**Gender**		
Male	130	34.85
Female	243	65.15
**Professional occupation**		
Health Professional	59	15.8
Non-health Professional	128	34.3
Student	129	34.6
Non-worker	57	15.3
**Qualifications**		
Elementary School Diploma	2	0.5
Middle School Diploma	36	9.6
High School Diploma	178	42.1
University Degree	157	47.7
**Diagnosis of Chronic Pathology (Diabetes, Hypertension, Chronic Bronchitis, CHD)**		
Yes	55	14.75
No	318	85.25
**Suffering from Gastrointestinal Diseases (GERD, Colitis, IBS, Gastritis)**		
Yes	136	36.5
No	237	63.5
**Vomiting and/or Diarrhoea in the past 3 months**		
Never	254	68.1
Once	86	23.1
More than once	33	8.8
**Cohabitants with Vomiting and/or Diarrhoea in the past month**		
Never	278	74.5
At least once	95	25.5

**Table 2.** Univariate analysis on variables associated with knowledge, attitudes and practice scores.

	OVERALL SCORE (maximum = 19)		KNOWLEDGE SCORE (maximum = 6)		ATTITUDES SCORE (maximum = 8)		PRACTICES SCORE (maximum = 5)	
	Median	p-value	Median	p-value	Median	p-value	Median	p-value
**Age**								
18–29	10	0.048	3	0.009	5	0.005	2	0.085
30–39	11		3		5		2	
40–49	11		3		5		3	
50–59	11		3		5		3	
60–69	11		2		6		3	
70–79	14		4		6		4	
**Gender**								
Male	11	0.772	3	0.187	5	0.995	3	0.746
Female	11		3		5		3	
**Professional occupation**								
Health Professional	13	<0.001	4	<0.001	6	<0.001	3	<0.001
Non-health Professional	10		3		5		2	
Student	10		3		5		2	
Non-worker	11		3		6		3	
**Qualifications**								
Elementary School Diploma	11	0.014	3	<0.001	4	0.015	4	0.300
Middle School Diploma	10		2		5		3	
High School Diploma	10		3		5		2	
University Degree	12		4		5		3	
**Diagnosis of Chronic Pathology (Diabetes, Hypertension, Chronic Bronchitis, CHD)**								
Yes	12	0.022	3	0.765	6	0.005	3	0.014
No	10		3		5		2	
**Suffering from Gastrointestinal Diseases (GERD, Colitis, IBS, Gastritis)**								
Yes	11	0.534	3	0.389	5	0.130	3	0.425
No	10		3		5		3	
**Vomiting and/or Diarrhoea in the past 3 months**								
Never	11	<0.001	3	0.770	5	<0.001	3	<0.001
Once	10		3		5		2	
More than once	9		3		4		2	
**Cohabitants with Vomiting and/or Diarrhoea in the past month**								
Never	11	<0.001	3	0.913	5	0.135	3	<0.001
At least once	10		3		5		2	

Table 3 contains the results of the multinomial logistic regression analysis on variables associated with the risk of suffering from vomiting/diarrhoea in the previous three months among participants. The onset of foodborne transmitted infections in participants was statistically significantly associated with a higher risk amongst 18−29 age group and among who had a previous gastrointestinal disease; contrarywise, Practices Score could be considered as a protective factor, as the adjusted odds ratio diminishes per each unit increment.

**Table 3.** Multinomial logistic regression on factors involved in the risk of foodborne transmitted infections.

	One FBD in the previous 3 months adjOR*	More than one FBD in the previous 3 months adjOR*
Score Practices (per unit increment)	0.79 (0.67–0.93)^b^	0.75 (0.59–0.96)^a^
Age, 18–29 (ref. >29 years old)	2.31 (1.37–3.89)^b^	3.43 (1.54–7.66)^b^
Presence of previous gastrointestinal disease (ref. No)	2.71 (1.6–4.58)^c^	2.7 (1.26–5.78)^a^

*Note: p-value: ^a^ <0.05, ^b^ <0.01 and ^c^ <0.001.*Adjusted for sex, qualifications, professional occupation, presence of chronic disease, knowledge score and attitudes score.

Table 4 shows the results of the logistic regression analyses on factors involved in the risk of suffering from vomiting/diarrhoea in the previous month among participants' cohabitants. Being male was statistically significantly associated with a risk, whereas a higher Practices Score was associated with a decrease in the risk of the onset of a FBD among the cohabitants of participants.

**Table 4.** Logistic regression on factors involved in the risk of foodborne transmitted infections among cohabitants.

	At least one FBD in the previous month adjOR*	p-value
Score Practices (per unit increment)	0.72 (0.62–0.85)	<0.0001
Sex, male (ref. female)	1.70 (1.04–2.77)	0.034

*Note: Adjusted for age, qualifications, professional occupation, presence of chronic disease, presence of gastrointestinal diseases, knowledge score and attitudes score.

## 4. Discussion

The aim of this study was to provide empirical data about food handling behaviours as well as perceptions of food safety at home through a survey focused on knowledge, attitudes, and practices questions. For this purpose, a KAP survey was administrated via snowball sampling approach to a total of 373 subjects resident in Sicily, Italy.

Foodborne transmitted diseases represent an important Public Health problem mainly due to their spread even in the most industrialized countries. In the past years, several epidemiologists overwatched FBDs, and this helped to estimate the extent of foodborne diseases and food-related diseases in the industrialized countries [39].

As previously cited, WHO estimated a foodborne disease burden of 12 DALYs per 100,000 subjects [9],[10]. A such high burden of disease could be confirmed by the present survey in which about 30% of the participants stated to have suffered from gastrointestinal disease in the previous three months. In this sense, the Institute of Health in Italy pointed out that 55% to 75% of all outbreaks occur inside households [40].

This study confirms some of the world evidences in the field of food safety. Nevertheless, the average levels of knowledge, attitudes and practices (score 3 out of 6, 5 out of 8 and 3 out of 5, representing respectively 50%, 62.5% and 60% of the maximum score) in Sicily were relatively lower than those found in other recently-carried out surveys [12],[41]. These results are somewhat of a concern for these populations, although it should be noted that it is difficult to make comparisons between surveys that used different questionnaires.

Moreover, it has been observed that the higher the Practices Score, the lower was the risk of getting an FBD among both participants and their cohabitants. This demonstrates that good practices could lead to a safer food consumption. The surprising evidence is that knowledge and attitudes are not significantly correlated with the risk of foodborne infections. Moreover, knowledge was not strongly correlated with practices, suggesting that having an information on this topic can be not enough for assuring to translate it into practice. Similar results, with low correlations between knowledge, attitudes and practices, have been also observed in another survey carried out in 2015 in Palestine [42]. This should be considered since several institutions suggest implementing educational campaigns and training for the public, but it is important to understand the knowledge can be not enough for significantly improve the food safety at home. It is also possible that knowledges are not enough for reducing the risk since the population has difficulties in perceiving the risk in domestic food preparation and consumption [43],[26],[29].

Suggestion that comes from our data is to implement good practices in the handling, conservation, and consumption of food, without neglecting knowledge and attitudes. It is essential to understand, however, that in the absence of a good degree of established practices, the risk of gastrointestinal signs and/or symptoms, and thus disease, is quite high. Furthermore, our study seems to suggest other risk factors: the young age (18−29 years) and the presence of previous intestinal diseases increase the risk of FBDs onset in the analysed sample, while male sex seems to be a risk factor for the onset of FBDs in the cohabitants of the participants who responded to the survey.

Finally, it should be noted that our study could have several limitations that should be considered. A first possible limit is due to the relatively small sample size; a self-selection bias should also be considered because of “snowball sampling” technique: given that this is a non-probability sampling technique, selected sample was mainly based on researcher friends' network and, therefore, that could not represent Sicilian population.

In fact, our sample was composed mainly of young people; for instance, 48% were aged between 18 and 29 years old whereas the average Sicilian population in 2019 had a mean age of 43.9 years. In addition, most of the participants (70%) were students and non-health professional: for these reasons, the survey sample could be not representative of the general population. Another limitation was that foodborne illnesses records were self-reported and without confirmation from medical, laboratory or epidemiological sources, and thus could not reflect the realistic level of foodborne disease within the country. However, it should be also stated that, although not fully representative, our results could be useful for confirming the role of unsafe home practices in determining the risk for foodborne infections. A final limitation is the lack of information about the frequency of consumption of meals at commercial establishments, which to the extent they occurred, could have played protective role in the risk of foodborne transmitted diseases. However, we note that the survey was carried out during the third COVID-19 epidemic and, therefore, there would have been far fewer respondents than usual who would have eaten outside of their residences. Nevertheless, we are confident that some of the KAPs in home settings would also apply in outdoor settings (for instance, hand washing habits, and food handling practices).

## 5. Conclusions

Our results confirm that foodborne disease could be strongly associated with food handling at home and with unsafe practices. This association is not influenced by knowledge and these latter are not correlated with practices nor foodborne infection risk. Specific education on food safety could help to reduce this risk but it is of paramount importance to remember that the adoption of good practices of food manipulation is the real key to assure a reduction in food outbreaks in residences.
